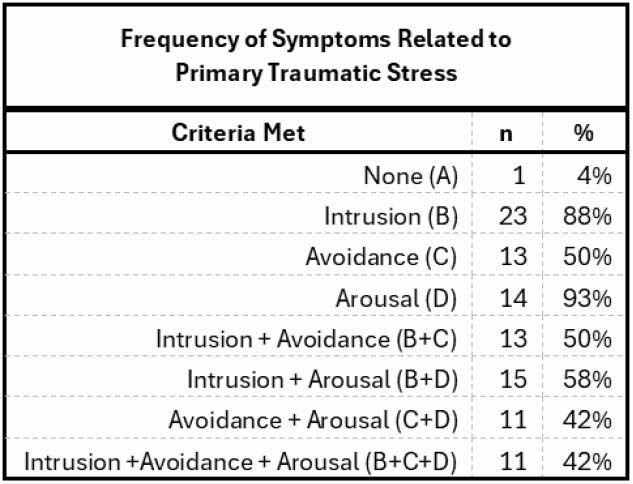# 944 Unmasking Primary Trauma in Burn Units

**DOI:** 10.1093/jbcr/iraf019.475

**Published:** 2025-04-01

**Authors:** Ashley Honea, W Michelle Spencer, Claudia Islas, Karen Richey, Kevin Foster

**Affiliations:** Diane & Bruce Halle Arizona Burn Center; Diane & Bruce Halle Arizona Burn Center; Diane & Bruce Halle Arizona Burn Center; Diane & Bruce Halle Arizona Burn Center; Diane & Bruce Halle Arizona Burn Center

## Abstract

**Introduction:**

The alarming rise in verbal and physical aggression toward healthcare workers has created an environment where those dedicated to healing face escalating threats to their psychological wellbeing, and physical safety. Direct exposure to these traumatogenic events can result in Primary Traumatic Stress (PTS) which may lead to Post Traumatic Stress Disorder (PTSD). These stressors can be further compounded by increased workloads, inadequate resources and a real or perceived lack of support from hospital leadership. Those working in the burn field may be at increased risk due to the inherent acute and long-term psychological stressors associated with the treatment of burn injuries. The specific dynamics of PTS and its progression to PTSD among burn care providers is underexplored. The purpose of this study was to determine the prevalence of symptoms indicative of primary stress and portents to the development of PTSD.

**Methods:**

The Secondary Traumatic Stress Scale is a 17-item scale that evaluates the frequency of symptoms of stress. A 5-point Likert scale is used to score responses for each item. While not developed specifically to measure PTS the subscales of intrusion, avoidance and arousal are used across multiple instruments. Scores were summed for items within each subscale, a score of ≥ 3 was considered positive for the individual item. The frequency of criteria was calculated. Exposure and duration of symptoms were not collected. The anonymous survey was distributed to burn center staff via RedCap.

**Results:**

There was a total of 26 respondents, only 1 did not meet criteria across all three domains. Intrusion criteria (5 items, possible range 5-25) mean score 12.5 (± 3.932). Avoidance criteria (7 items, possible range 7-35) mean score 16.42 (±5.247). Arousal criteria (5 items, possible range 5-25) mean score 12.38 (±13.105). The possible score range for the full scale was 17-85 and respondents averaged 41.31 (±13.105).

**Conclusions:**

Overall response rate was low and may indicate those who were experiencing symptoms were more likely to respond. Still, this study highlights the prevalence of PTS symptoms in burn staff. The repeated exposure to traumatic events in burn care may lead to the development of post-traumatic stress disorder (PTSD) among staff. Future studies are needed to determine the magnitude of this problem so that data-driven solutions can be implemented. Initiatives such as resilience training programs focusing on emotional regulation, stress management, and adaptive coping strategies specifically designed for burn care environments would likely be a great benefit. Such initiatives could potentially foster a resilient workforce capable of maintaining high-quality care amidst the unique challenges of burn healthcare settings.

**Applicability of Research to Practice:**

This study highlights the need for enhanced education and interventions related to mitigating primary stress exposure in the burn care environments.

**Funding for the Study:**

N/A